# Diagnostic accuracy of sonography for pleural effusion: systematic review

**DOI:** 10.1590/S1516-31802010000200009

**Published:** 2010-03-04

**Authors:** Alexandre Grimberg, David Carlos Shigueoka, Álvaro Nagib Atallah, Sergio Ajzen, Wagner Iared

**Affiliations:** I MD. Radiologist in the Department of Diagnostic Imaging, Universidade Federal de São Paulo — Escola Paulista de Medicina (Unifesp-EPM), São Paulo, Brazil.; II PhD. Professor in the Department of Diagnostic Imaging, Universidade Federal de São Paulo — Escola Paulista de Medicina (Unifesp-EPM), São Paulo, Brazil.; III PhD. Full professor of the Discipline of Emergency Medicine and Evidence-Based Medicine, Department of Medicine, Universidade Federal de São Paulo, Escola Paulista de Medicina (Unifesp-EPM), São Paulo, Brazil.; IV PhD. Full professor of the Department of Diagnostic Imaging, Universidade Federal de São Paulo — Escola Paulista de Medicina (Unifesp-EPM), São Paulo, Brazil.; V MD. Radiologist in the Department of Diagnostic Imaging and affiliated researcher at the Brazilian Cochrane Center, Universidade Federal de São Paulo — Escola Paulista de Medicina (Unifesp-EPM), São Paulo, Brazil.

**Keywords:** Pleural effusion, Ultrasonography, Sensitivity and specificity, Review [Publication type], Meta-analysis [Publication type], Diagnostic imaging, Derrame pleural, Ultrassonografia, Sensibilidade e especificidade, Revisão, Metanálise, Diagnóstico por imagem

## Abstract

**CONTEXT AND OBJECTIVE:**

The initial method for evaluating the presence of pleural effusion was chest radiography. Isolated studies have shown that sonography has greater accuracy than radiography for this diagnosis; however, no systematic reviews on this matter are available in the literature. Thus, the aim of this study was to evaluate the accuracy of sonography in detecting pleural effusion, by means of a systematic review of the literature.

**DESIGN AND SETTING:**

This was a systematic review with meta-analysis on accuracy studies. This study was conducted in the Department of Diagnostic Imaging and in the Brazilian Cochrane Center, Discipline of Emergency Medicine and Evidence-Based Medicine, Department of Medicine, Universidade Federal de São Paulo (Unifesp), São Paulo, Brazil.

**METHOD:**

The following databases were searched: Cochrane Library, Medline, Web of Science, Embase and Literatura Latino-Americana e do Caribe em Ciências da Saúde (Lilacs). The references of relevant studies were also screened for additional citations of interest. Studies in which the accuracy of sonography for detecting pleural effusion was tested, with an acceptable reference standard (computed tomography or thoracic drainage), were included.

**RESULTS:**

Four studies were included. All of them showed that sonography had high sensitivity, specificity and accuracy for detecting pleural effusions. The mean sensitivity was 93% (95% confidence interval, CI: 89% to 96%), and specificity was 96% (95% CI: 95% to 98%).

**CONCLUSIONS:**

In different populations and clinical settings, sonography showed consistently high sensitivity, specificity and accuracy for detecting fluid in the pleural space.

## RESUMO

**CONTEXTO E OBJETIVOS:**

O método inicial para a avaliação da presença dos derrames pleurais foi a radiografia de tórax. Estudos isolados demonstraram que a ultrassonografia apresenta uma acurácia maior que a radiografia para este diagnóstico, entretanto, não se encontram disponíveis na literatura revisões sistemáticas sobre este tema. Assim, o objetivo deste estudo é avaliar a acurácia da ultrassonografia na detecção de derrame pleural, por meio de revisão sistemática da literatura.

**TIPO DE ESTUDO E LOCAL:**

Revisão sistemática com metanálise de estudos de acurácia. O estudo foi conduzido no Departamento de Diagnóstico por Imagem e no Centro Cochrane do Brasil/Disciplina de Medicina de Urgência e Medicina Baseada em Evidências do Departamento de Medicina, Universidade Federal de São Paulo (Unifesp), São Paulo, Brazil.

**MÉTODOS:**

Foram pesquisadas as seguintes bases de dados: Cochrane Library, Medline, Web of Science, Embase e Literatura Latino-Americana e do Caribe em Ciências da Saúde (Lilacs). As referências de estudos relevantes foram verificadas para adicionais citações de interesse. Foram incluídos estudos nos quais a acurácia da ultrassonografia para a detecção de derrame pleural tenha sido testada, com padrão referência aceitável (tomografia computadorizada ou drenagem torácica).

**RESULTADOS:**

Foram incluídos quatro estudos. Todos apresentaram alta sensibilidade, especificidade e acurácia para a detecção de derrame pleural pela ultrassonografia. A sensibilidade média foi de 93% (intervalo de confiança, IC 95%: 89% a 96%), e a especificidade 96% (IC 95%: 95% a 98%).

**CONCLUSÕES:**

A ultrassonografia, em diferentes populações e cenários clínicos, apresentou consistentemente alta sensibilidade, especificidade e acurácia na detecção de líquido no espaço pleural.

## Introduction

The initial method for evaluating pleural effusions was thoracic radiography. For orthostatic posteroanterior (PA) radiographs, a minimum of 175 ml of pleural fluid is needed for detection.^1^ Lateral radiographs allow diagnosis with volumes starting from 75 ml, since the fluid tends to first accumulate in the posterior portions of the costophrenic recess.^1^ Radiographs in the supine position show lower sensitivity, and sometimes large effusions can be missed if they are bilateral.^2^ The main sign that leads to a diagnosis of pleural effusion from supine radiographs is greater opacity of the hemithorax, with no blurring of vascular structures.^2^

The view that is most sensitive for detecting fluid in the pleural space is lateral decubitus with horizontal rays, which can detect effusions starting at 5 ml.^3^ However, not all patients can undergo this radiographic view, especially patients in intensive care units (ICUs) and trauma victims in the emergency room. Some technical issues can further limit the quality of the radiographs produced when the patient is on a bed, such as movement of the thoracic wall, patient rotation and supine position with the film placed behind the patient and short focus-film distance.^4^

Sonography has been used to detect pleural effusions since the late 1960s.^5^ A study in 1976, using A-mode sonography to detect pleural effusion found sensitivity of 93%.^6^ Other studies have been conducted over the years, many of them comparing the sensitivity of sonography and radiography, and better results have been shown with sonography. However, the use of sonography is not as widespread as the use of radiography for this purpose.

In comparison with sonography, radiography has the advantage of evaluating the skeleton, as well as the lung parenchyma and the mediastinum.^7^ Moreover, radiography allows assessment of thoracic tubes and catheters.^5^ On the other hand, sonography has the capacity to clarify the nature of opaque lesions such as effusions, atelectasis, masses and consolidations.^8^

Over the years, sonography has been restricted to detection of effusions and procedure guidance (thoracocentesis, biopsy or drainage),^2^ providing a high success rate and low morbidity^5^. In recent years, studies have shown that sonography achieves better results than radiography in measuring the effusion volume.^8-11^ However, the confidence intervals for such measurements remain wide. For this reason, and because of the heterogeneity of the methods proposed in different studies, large-scale use of sonography has been impeded.

Computed tomography is considered to be the gold standard for detection of pleural effusions. In addition to enabling evaluation of the pleural space, it allows accurate and detailed evaluation of the thoracic wall, lung parenchyma and mediastium.^12-14^ Its limitations are its low availability in remote centers, high cost and high radiation dose, and the need to take patients to the examination room, which delays the diagnosis.^4,5,12-14^ This last item is particularly important in relation to seriously ill patients or trauma victims.

Among radiologists, sonography is widely recognized as a sensitive and specific method for detection of pleural effusion. This view is not widely held among the remainder of the medical community, perhaps because the literature does not provide the maximum level of evidence. Such evidence would consist of a systematic review of multicenter high-quality homogeneous accuracy studies.

Sonography is a fast, portable low-cost method that does not use ionizing radiation, and there are indications that it is highly accurate for detection of pleural effusion. If the level of evidence regarding such accuracy could be raised, the use of sonography as the first-choice method for evaluating patients with suspected pleural effusion could spread.

## Objective

The aim of this study was to evaluate the accuracy of sonography for detecting pleural effusion through a systematic review of the literature, which represents the highest level of evidence for evaluations on the accuracy of a diagnostic test.

### Method

We conducted a systematic review with meta-analysis on diagnostic accuracy studies. Studies in English, Spanish or Portuguese that evaluated the accuracy of sonography for detecting pleural effusions were included. The following reference standards were considered acceptable: computed tomography and thoracic drainage. Studies that did not use an acceptable reference standard were excluded.

The study was conducted in the Department of Diagnostic Imaging and in the Brazilian Cochrane Center, Discipline of Emergency Medicine and Evidence-Based Medicine, Department of Medicine, Universidade Federal de São Paulo (Unifesp), São Paulo, Brazil.

### Search strategy

Studies were retrieved from the following sources: PubMed (1966 to October 2008), Excerpta Medica Database (Embase) (1980 to January 2009), Web of Science (to October 2008) and Literatura Latino-Americana e do Caribe em Ciências da Saúde (Lilacs) (1982 to October 2008). A general search strategy was used, which was adaptable to the characteristics of each database, in order to identify studies containing the words and subject headings "sonography" and "pleural effusion". Relevant study references were also screened for additional potential studies.

#### Medline strategy via PubMed:

((Pleural Effusion) OR (Effusion, Pleural) OR (Effusions, Pleural) OR (Pleural Effusions)) AND ((Ultrasonic Imaging) OR (Imaging, Ultrasonic) OR (Imagings, Ultrasonic) OR (Ultrasonic Imagings) OR (Sonography, Medical) OR (Medical Sonography) OR (Echography) OR (Echotomography) OR (Echotomographies) OR (Echotomography, Computer) OR (Computer Echotomography) OR (Tomography, Ultrasonic) OR (Ultrasonic Tomography) OR (Diagnosis, Ultrasonic) OR (Diagnoses, Ultrasonic) OR (Ultrasonic Diagnoses) OR (Ultrasonic Diagnosis))

#### Lilacs strategy:

"Derrame Pleural" OR "Pleural Effusion" [Palavras] and Ultrasonography OR Sonography OR Ultra-sonografia OR Ultrassonografia OR Ultrassom OR Ultra-som OR Ultrasonido OR Ultrasonografía [Palavras]

"Derrame Pleural" OR "Pleural Effusion" [Palavras] and Ultrasonography OR Sonography OR Ultra-sonografia OR Ultrassonografia OR Ultrassom OR Ultra-som OR Ultrasonido OR Ultrasonografía [Palavras] or ("ULTRA-SONOGRAFIA") AND "DERRAME PLEURAL" [Descritor de assunto]

#### Web of Science strategy:

((Pleural Effusion) OR (Effusion, Pleural) OR (Effusions, Pleural) OR (Pleural Effusions)) AND Topic=((Ultrasonic Imaging) OR (Imaging, Ultrasonic) OR (Imagings, Ultrasonic) OR (Ultrasonic Imagings) OR (Sonography, Medical) OR (Medical Sonography) OR (Echography) OR (Echotomography) OR (Echotomographies) OR (Echotomography, Computer) OR (Computer Echotomography) OR (Tomography, Ultrasonic) OR (Ultrasonic Tomography) OR (Diagnosis, Ultrasonic) OR (Diagnoses, Ultrasonic) OR (Ultrasonic Diagnoses) OR (Ultrasonic Diagnosis))

#### Embase strategy:

((Pleural Effusion) OR (Effusion, Pleural) OR (Effusions, Pleural) OR (Pleural Effusions)) AND ((Ultrasonic Imaging) OR (Imaging, Ultrasonic) OR (Imagings, Ultrasonic) OR (Ultrasonic Imagings) OR (Sonography, Medical) OR (Medical Sonography) OR (Echography) OR (Echotomography) OR (Echotomographies) OR (Echotomography, Computer) OR (Computer Echotomography) OR (Tomography, Ultrasonic) OR (Ultrasonic Tomography) OR (Diagnosis, Ultrasonic) OR (Diagnoses, Ultrasonic) OR (Ultrasonic Diagnoses) OR (Ultrasonic Diagnosis)

One reviewer evaluated all the titles and summaries of the articles encountered (AG). For all articles that potentially met the inclusion criteria, and for indeterminate articles, the full text was assessed. Two reviewers evaluated these selected articles, and they extracted data independently (AG, WI). Ambiguous cases were resolved by reaching a consensus in the presence of a third reviewer (DCS). Data were evaluated using the Review Manager program (RevMan), version 5.0.20, in order to obtain sensitivity and specificity values and the respective 95% confidence intervals. Weighted averages were used to express overall sensitivity and specificity.

The quality evaluation on the studies was conducted using QUADAS (Quality Assessment of Diagnostic Accuracy Studies).^15^ The following eight relevant questions taken from QUADAS were applied:

1. Was the patient spectrum representative of patients seen in actual clinical practice?
2. Was the reference standard able to identify the target condition correctly?
3. Was the period of time between the index test and the reference standard short enough, so that it was unlikely that the target condition had been modified between the two tests?
4. Were all patients or just a randomly selected subgroup evaluated with the reference standard?
5. Did all patients receive the same reference standard, in spite of the index test results?
6. Were the results from the index test interpreted without knowledge of the reference standard results?
7. Were the results from the reference standard interpreted without knowledge of the index test results?
8. Were the causes of exclusions of cases explained?

## Results

### Study selection

1,187 titles were found in the Medline database, via PubMed, 963 titles in Embase, 43 in the Web of Science and 10 in Lilacs. One additional article was found through checking the relevant references. There were many studies that were indexed in more than one database. After excluding duplications, 13 studies showed potential for inclusion and were retrieved.

**Figure 1.**
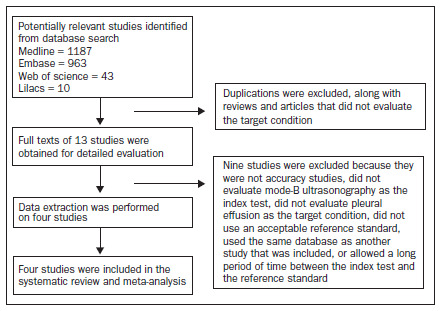
Flow diagram of the study inclusion process.

Among these, four met all the inclusion criteria and did not meet any exclusion criteria. **Figure 1** summarizes the search strategy and the process of study selection.

The other nine were excluded because of the following reasons, as shown in **Figure 1**:

- They were not accuracy studies (two studies).
- The reference standard was sonography and the index test was radiography (two studies).
- The target condition was not pleural effusion (one study).
- The reference standard was not acceptable (one study).
- The study used the same database as another study already included (one study).
- The index test was not B-mode sonography, the reference standard was not described and it was not possible to extract data to build the 2 × 2 table (one study).
- The reference standard used for some of the subjects was not acceptable; and the period of time between the index test and the reference standard was not short enough (one study).

The study by Gryminsky et al.^6^ was excluded because it used A-mode sonography instead of B-mode. The reference standard was not described and only data referring to sensitivity were reported. Specificity was not mentioned. In both studies by Kocijancic et al.^16,17^ the reason for exclusion was that the index test was radiography and the reference standard was sonography. The study by Ma et al.^18^ was excluded because it used the same sample of patients (same database) as the study by Ma and Mateer.^7^ The study by Reissig et al. was excluded for two reasons: the target condition was pneumothorax and hydropneumothorax, and the reference standard test (computed tomography) was not performed in all patients. The reference standard (radiography) was not acceptable in two studies that were also excluded: Rozycki et al.^5^ and Sisley et al.^19^ In the case of Rozycki et al.,^5^ the period of time between the index test and the reference standard was not short enough (sometimes over 24 hours), and this constituted another reason for exclusion. Both Tayal et al.^20^ and Yu et al.^21^ researches were not accuracy studies and they were excluded because of that. In Tayal et al.,^20^ The results consisted of changes to the probability rate (based on subjective evaluation made by the attending physician, without description of the reference standard), and of changes to case management. In Yu et al.,^21^ the results consisted of descriptions of how chest sonography changed the diagnosis based on radiography, and of changes to case management.

### Patient spectrum

The studies included evaluated different groups of patients in specific clinical settings. Ma et al.^7^ studied the accuracy of sonography in the emergency room, in relation to trauma victims. Kataoka and Tanaka^12^ studied patients with exacerbation of stable chronic heart failure or acute heart failure. Lichtenstein et al.^4^ studied patients in the ICU hospitalized because of acute respiratory distress syndrome (ARDS). Rocco et al.^14^ studied trauma victims with internal injuries only, under mechanical ventilation, also hospitalized in the ICU.

### Technical differences among the studies

There were technical differences among the studies, regarding the sonographic examination. Ma et al.^7^ used a sonographic window consisting of the lateral thoracic walls during focused assessment with sonography for trauma (FAST). Kataoka and Tanaka^12^ examined the patients in a seated position, under conditions of shallow breathing, giving emphasis to the posterolateral thoracic wall, often guided by the liver, spleen and kidney locations. Lichtenstein et al.^4^ divided the thorax of each patient into 12 regions and studied each region individually, using both the supine and the lateral position. Rocco et al.^14^ also divided the thorax into 12 regions, but always performed the examination in the supine position, because of the patients’ clinical conditions.

### Quality of the studies included

Three studies used computed tomography as the reference standard, which was considered ideal.^4,12,14^ One study used computed tomography and thoracic drainage.^7^

One study performed the index test immediately after performing the reference standard.^4^ In another study,^12^ the time interval was shorter than two hours in 68% of the cases; shorter than 12 hours in 20% and shorter than 24 hours in 12%. One study^7^ did not give such information, and one stated that the maximum time interval was one hour.^14^

**Figure 2.**
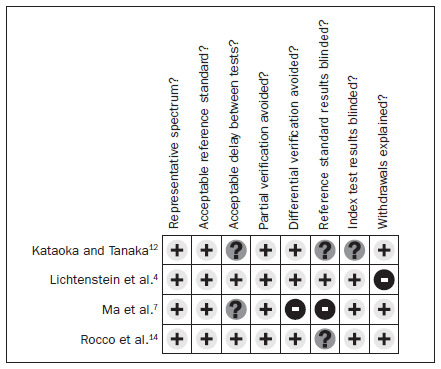
Graphic representation of the evaluation on the quality of the studies included.

One study^7^ used different reference standards (computed tomography and thoracic drainage). Only one study^4^ described blinding for the reference standard. Two studies^12,14^ did not make the blinding clear and one^7^ stated that there was no blinding and that the reference standard was interpreted with previous knowledge of the sonography results. Three studies^4,7,14^ blinded the interpreters of the index test, while one did not make it clear whether there was any blinding. Regarding exclusions and withdrawals, only one of the studies^4^ did not cite them, while the other three cited and explained them. **Figure 2** summarizes the quality of the studies evaluated.

### Evaluation of the accuracy of sonography for detecting pleural effusion

**Figure 3.**
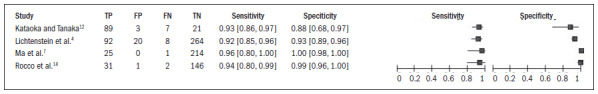
Forest plot for the accuracy of chest sonography in detecting pleural effusions (Kataoka and Tanaka^12^: n = 120; Lichtenstein et al.^4^: n = 384; Ma et al.^7^: n = 240; Rocco et al.^14^: n = 180; Total: n = 924).

**Figure 4.**
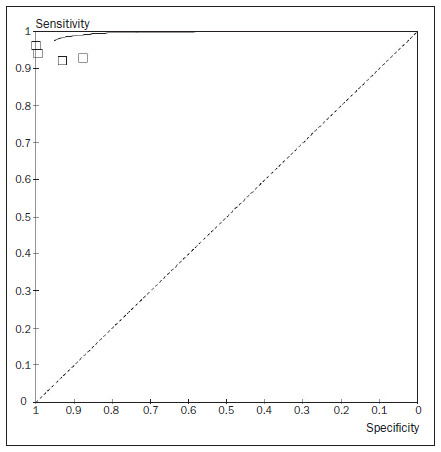
Summary receiver operating characteristic (ROC) curve for the accuracy of sonography in detecting pleural effusion.

**Figure 5.**
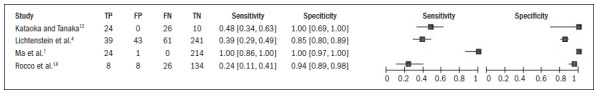
Forest plot for the accuracy of chest radiography in detecting pleural effusions.

All the studies showed high sensitivity, specificity and accuracy in relation to detecting pleural effusion through sonography. The average sensitivity was 93% (95% confidence interval, CI: 89% to 96%) and the average specificity was 96% (95% CI: 95% to 98%). **Figures 3, 4 and 5** summarize the results found.

## Discussion

Although we found heterogeneity in the spectrum of patients, the target condition was always the same: pleural effusion. Regardless of its cause, the accuracy of sonography in detecting the target condition was similar in all populations. For this reason we considered it appropriate to group the studies in a meta-analysis. Even among seriously ill patients who were difficult to move, and even in the setting of emergency attendance of trauma, sonography showed high sensitivity and specificity. It also showed high inter-observer agreement in the study that evaluated this parameter.^22^

Among the limitations on the use of sonography for detecting pleural effusion, we can highlight the difficulties in using it for obese patients, patients with subcutaneous emphysema and patients whose entire chest surface cannot be examined using the transducer, because of wounds, bandages or catheters.^4^

It is also important to highlight the need to train the professionals involved in performing the examination.^7,20^ Learning the specific technique for detecting pleural effusion is not very complex, and this can be properly achieved in a short period of time, not only by surgeons and clinicians, but also by students with no previous experience of the method.^4,5,14^

Concerning the duration of the examination, which is a critical point for sonography performed in emergency room settings, the studies that evaluated this parameter showed that the examinations were performed satisfactorily in a few minutes, thus making it possible to use sonography in these situations.^14,20^

All four studies included in this review also investigated the accuracy of radiography for detecting pleural effusion.^4,7,12,14^ One of these studies found similar results for both methods, and all the other three showed significantly better results for sonography. For radiography, the sensitivity for detecting pleural effusion ranged from 24% to 100% and the specificity ranged from 85% to 100%.

## Conclusion

Sonography, which is a portable low-cost radiation-free method, showed consistently high sensitivity, specificity and accuracy in detecting fluid in the pleural space, in different populations and clinical settings.
